# Acalculous cholecystitis occurring in the context of *Plasmodium malariae* infection: a case report

**DOI:** 10.1186/1752-1947-7-197

**Published:** 2013-07-26

**Authors:** Eleanor F Harris, Eugenie Younger, Meirion B Llewelyn

**Affiliations:** 1 Department of Infectious Diseases, Department of Medicine, Royal Gwent Hospital, Newport, Wales NP20 2UB, United Kingdom

**Keywords:** Acalculous cholecystitis, Malaria, *Plasmodium malariae*

## Abstract

**Introduction:**

Acalculous cholecystitis has been shown to occur in the context of malarial infection with *Plasmodium vivax* and *Plasmodium falciparum* and requires prompt diagnosis and treatment to prevent complications. To the best of our knowledge this is the first reported case of the disease occurring in a patient infected with *Plasmodium malariae.*

**Case presentation:**

We report the first case of acalculous cholecystitis associated with *Plasmodium malariae* in a 59-year-old male Nepalese ex-Gurkha soldier. He presented with fever and vomiting and later developed right upper quadrant pain. Abdominal ultrasound and computed tomography scans confirmed acalculous cholecystitis for which he was treated medically with chloroquine, gentamicin and metronidazole. He made a full recovery.

**Conclusions:**

Physicians should be aware that in addition to *Plasmodium vivax* and *Plasmodium falciparum* infections, acalculous cholecystitis can occur in the context of *Plasmodium malariae* infection. Mechanisms for this are discussed but further studies are needed to establish the etiology of this association.

## Introduction

Acalculous cholecystitis (or acute acalculous cholecystitis; AAC) is a condition characterized by gallbladder inflammation without gallstones, usually diagnosed by ultrasound. It is most commonly attributed to severe physiological insult such as sepsis, burns or trauma [1] but there have been several reports linking the disease with malaria infection [2,3]. Of the five *Plasmodium* parasites responsible for the disease in humans (*Plasmodium falciparum, Plasmodium vivax, Plasmodium malariae, Plasmodium ovale* and *Plasmodium knowlesi),* only *P. falciparum* and *P. vivax* have so far been associated with AAC. We report the first case of AAC occurring in the context of *P. malariae* infection.

## Case presentation

A 59-year-old male Nepalese ex-Gurkha soldier currently working in Ghana was admitted with a 3-day history of malaise, fever, rigors and vomiting. He had returned from a trip to Nepal 3 weeks previously. He had a history of two previous malarial infections; the last occurring 1 year prior to admission. He had never used malarial prophylaxis. The only other significant medical history was of gout.

On admission he was alert and orientated, had a temperature of 38.3°C, pulse 70 beats/minute and blood pressure of 106/64mmHg. His physical examination was otherwise unremarkable. Laboratory studies showed a white blood cell count of 7.4 × 10^9^/L, hemoglobin 13.6g/dL and platelets 94 × 10^9^/L (normal: 150 to 400 × 10^9^/L). Total bilirubin was 50μmol/L (1 to 21μmol/L) and conjugated bilirubin 16μmol/L (0 to 7μmol/L). Alanine transaminase (ALT) was 63U/L (0 to 59U/L) and alkaline phosphatase (ALP) 118U/L.

An examination of the blood film showed that malarial parasites were present and subsequently confirmed by the London School of Tropical Medicine as *Plasmodium malariae.* He was therefore treated with oral chloroquine in line with the Health Protection Agency policy. A day after admission he developed generalized abdominal pain, followed in 24 hours by right upper quadrant (RUQ) pain. At this stage he had a persistent fever and laboratory studies demonstrated a rising C-reactive protein and deranged liver function tests. The peak value of ALP was 185U/L (30 to 130U/L) and ALT 103U/L (0 to 59U/L).

Abdominal computed tomography (CT) showed thickening of the gallbladder wall with surrounding fluid suspicious of cholecystitis. An ultrasound examination also showed gallbladder thickening (>3.5mm) and pericholecystic fluid, thus fulfilling the necessary criteria needed for a diagnosis of acalculous cholecystitis [4] (Table 1). Mild splenomegaly was also noted on ultrasound. There was no evidence of gallstones. The surgical team was involved and because the liver function tests were only mildly deranged and there was no radiological evidence of duct dilatation, further imaging was deemed unnecessary.

**Table 1:** Ultrasonography criteria for acute acalculous cholecystitis

**Major criteria**	**Minor criteria**
• Gallbladder wall thickening ≥3mm	• Gallbladder distension (>5cm)
• Wall edema	• Echogenic bile (sludge)
• Intramural gas	
• Mucosal sloughing	
• Pericholecystic fluid	
• Sonographic Murphy’s sign	

Two major or one major and two minor criteria are needed for diagnosis.

Antibiotic treatment with gentamicin and metronidazole was commenced and over the next 3 days his fever resolved and liver function tests returned to normal. Repeat blood film showed no evidence of malarial parasites. His symptoms had completely resolved by day 11 and he was discharged home. On review in clinic 6 weeks later he had made a full recovery.

## Discussion

Since the first reported case in 1999 [5] there have been 25 reported cases of AAC occurring in the context of malaria: 19 associated with *P. falciparum* infections; three associated with *P. vivax* (two in adults and one in a child), two cases involving a mixed infection with *P. vivax* and *P. falciparum* and our case associated with *P. malariae* infection as detailed above.

A recent review of 16 of these cases compared the presentation and clinical course of the illness across the cases [2]. Our case seems to correspond well with the clinical presentation of AAC in *P. falciparum* and *P. vivax* infections, namely nausea, vomiting and abdominal pain in association with mildly deranged liver function tests. Fever and moderate hypotension were also present in our case and these were common in other reported cases. As in the present case, RUQ abdominal pain was reported an average of 1 to 3 days after presentation.

The majority of the cases were diagnosed using ultrasound, although two also used hepatobiliary iminodiacetic acid (HIDA) scans [2]. Ultrasound is generally accepted as the best first line investigation of AAC due to availability and portability [6]. A recent review reported sensitivities and specificities for AAC diagnosis of up to 92% and 100% respectively, although there is a large amount of variance between studies [6]. CT has a similar accuracy to ultrasound and is useful when an alternative diagnosis is being considered [4]. HIDA scans, although accurate in detecting calculous cholecystitis, have been shown to have a sensitivity as low as 68% in the case of acalculous cholecystitis [7].

With regard to disease course, the relatively fast resolution of symptoms with medical management as described above was also seen in most of the other cases [2,3]. The recommended treatment of AAC is broad spectrum antibiotics to cover enterococci and Gram-negative bacilli [1]. This is followed by percutaneous cholecystostomy if medical management is unsuccessful and severe cases may require cholecystectomy [1]. Only five of the 25 total cases involved surgical management (in the form of percutaneous cholecystostomy) and these were all in severe cases of malaria, four with *P. falciparum* infections [3,8,9] and one with *P. vivax*[2]. Prompt diagnosis and treatment is important because delay can lead to gallbladder perforation or gangrene [1]. All cases of AAC reported with malaria infection had good outcomes however, with no deaths or significant residual morbidity reported.

The mechanisms underlying the occurrence of AAC in malaria infections are largely unknown with three main mechanisms being considered at present:

1. Ischemia

Oxygen-demand-related ischemia and subsequent reperfusion injury to the gallbladder are thought to be important factors in the development of AAC because the disease is seen in a number of low flow states and during critical illnesses such as in burns and septic shock [1]. Malaria can cause these low flow states due to severe hypotension and severe illness, in addition to hemolytic anemias which have been suggested as contributory factors [2]. However, only three of the reported cases of malaria-associated AAC described severe malaria [2,9,10], whereas most, as in the case of *P. malariae* above, were relatively mild malarial infections and thus do not strongly support the ischemia mechanism. It has been suggested that a combination of mild hypoperfusion and hemolytic anemia may contribute to oxygen-demand ischemia but this remains to be proved [2].

2. Cytokines

Another proposed mechanism to explain AAC associated with malaria infections is the imbalance of anti-inflammatory and pro-inflammatory cytokines which can occur during malarial infections. Cytokines such as interleukin (IL)-6, IL-10, IL-12 and tumor necrosis factor-alpha are known to be induced during *P. vivax* and *P. falciparum* infections [11,12] and may therefore create systemic inflammatory responses which can cause gallbladder injury. There are few studies of *P. malariae* pathogenesis, however, and the cytokine effects are unknown, although this remains a possibility for further study.

3. Sequestration

A third mechanism for the pathogenesis of AAC in malaria is the phenomenon of sequestration. This has been well described in *P. falciparum* infections and is the process whereby a protein specific to *P. falciparum* causes protrusions called “knobs” to form on infected erythrocytes which then adhere to vessel walls and each other. This results in widespread vascular sludging and ischemia [2]. This could then lead to AAC due to gallbladder ischemia.

However, this elegant explanation cannot apply in the same way to *P. vivax* or *P. malariae* infections because they have not been shown to produce this protein or cause sequestration in the same way [13,14]. Nonetheless, *P. vivax* has been shown to induce erythrocyte binding using endothelial receptors in a similar manner to *P. falciparum*[13] and thus this may still be a potential explanation for AAC in *P. vivax* infections. With regard to our case, no erythrocyte binding has been shown in *P. malariae* infections although a recent atomic force microscope image of an erythrocyte infected with *P. malariae* has revealed a high density of “spiky excrescences” on its surface [15]. Their function remains unknown at present and further research is needed before this can be suggested as a possible mechanism for AAC in this case.

These three mechanisms all offer plausible explanations for malaria-related AAC in specific circumstances, but none offer an overall convincing etiology which explains the situation seen with different plasmodium species and differing severities of malarial infection. Interestingly, one recent paper has suggested that AAC can occur as an idiopathic phenomenon in young healthy individuals raising the possibility that malaria and AAC are in fact unrelated events. However, the study was small (11 patients with AAC) and was a retrospective study looking at patients who had cholecystectomy for their AAC [16]. This is in contrast to the cohort of patients with AAC in the context of malaria infection because they were mainly treated medically. Nonetheless this is an important area to consider and needs further investigation.

## Conclusions

In addition to *P. falciparum* and *P. vivax* infection, AAC should be also considered a complication of *P. malariae* infection. The mechanisms underlying this are unclear and require further study. Clinical features and management are comparable to those in *P. falciparum* and *P. vivax* infections. In any case of malaria, regardless of the species involved, deranged liver function tests and RUQ pain should prompt investigations to exclude AAC in order to allow timely treatment if required.

## Consent

Written informed consent was obtained from the patient for publication of this case report. A copy of the written consent is available for review by the Editor-in-Chief of this journal.

## Competing interests

The authors declare that they have no competing interests.

## Authors’ contributions

EH and EY collected the case report data, analyzed the case and drafted the case report section. EH was the major contributor in writing the manuscript. ML was the consultant responsible for the care of the patient and provided the initial concept for the case report, as well as assistance in editing. All authors read and approved the final manuscript.
